# Metabolic equivalent of task (METs) thresholds as an indicator of physical activity intensity

**DOI:** 10.1371/journal.pone.0200701

**Published:** 2018-07-19

**Authors:** Márcio de Almeida Mendes, Inácio da Silva, Virgílio Ramires, Felipe Reichert, Rafaela Martins, Rodrigo Ferreira, Elaine Tomasi

**Affiliations:** 1 Post-Graduate Program in Epidemiology, Federal University of Pelotas, Pelotas, Brazil; 2 Physical Education School, Federal University of Pelotas, Pelotas, Brazil; 3 Sul-rio-grandense Federal Institute for Education, Science and Technology, Camaquã, Brazil; Sao Paulo State University - UNESP, BRAZIL

## Abstract

The purpose of the study was to identify and compare validity parameters of different absolute intensity thresholds in METs, using relative intensity classification as criterion measure. Convenience sampling was used to recruit total of 112 adults. The participants carried out an incremental maximal cycle ergometer test and asked to perform nine free-living activities. The oxygen uptake was measured by a *VO2000*^®^ gas analyser throughout the tests. The intensity thresholds were identified using Receiver Operator Characteristic (ROC) curve analysis, having relative intensity categories as criterion measure. A total of 103 participants attended the two visits. Among 54 men and 49 women, the mean (± SD) ages were 36.1 (± 11.1) and 33.9 (± 10.6) years, respectively. The intensity thresholds identified were 4.9 METs for moderate and 6.8 METs for vigorous physical activity. In conclusion, the physical activity thresholds, generated according to the entire sample, were higher and presented improved specificity when compared to thresholds currently recommended. Moreover, these parameters presented relatively high accuracy, even when applied to specific groups such as sex, age, nutritional status and physical fitness.

## Introduction

Physical activity is defined as any body movement resulting in energy expenditure higher than resting [1]. It might also be characterized as behaviour of complex assessment, considering its diversity regarding different body movements and dimensions such as frequency, intensity and duration.

There are several health benefits associated with regular physical activity practice [2] and these positive effects are not only related to the total energy expenditure, but also attributed to the intensities in which physical activity might be performed [3]. Therefore, it is essential to precisely determine physical activity intensities.

Currently, there are different proposals of thresholds based on relative intensities (considering individual characteristics) and absolute intensities (which do not take into account individual characteristics) [4]. Guidelines have recommended using metabolic equivalent of task (METs) as reference thresholds of absolute intensities (light, <3.0 METs; moderate, 3.0–5.9 METs; vigorous ≥6.0 METs) [3], however, its validity parameters are not available in the literature.

Misclassification of light, moderate or vigorous physical activity brings an important limitation for the study of this behaviour, since it may under or overestimate physical activity estimates and distort its associations with health outcomes. Although these thresholds of intensity have been widely applied in epidemiological research, it is crucial that their sensitivity and specificity parameters are properly evaluated. Thus, the aim of the present study was to identify and compare validity parameters of different absolute intensity thresholds in METs using relative intensities as criterion measure.

## Material and methods

### Sample

The study was carried out between April and September 2016 in a southern city of Brazil. Participants (112 adults) were recruited by convenience sampling through advertisement using various social outlets. The participants included varying fitness levels, ages, and gender to increase the representation of the population. Volunteers with chronic diseases (such as diabetes, cardiovascular or pulmonary diseases) were excluded from the study. Readiness for physical activity practice was assessed by the Physical Activity Readiness Questionnaire (PAR-Q) [5], excluding those potential participants presenting at least one positive answer. This study was approved by the Ethics Committee of the Medicine School—Federal University of Pelotas (UFPel), according to protocol number 1.258.787/2015. All participants voluntarily signed a written informed consent and they could abandon the study at any time.

### Measures

The data collection was performed in two visits to the laboratory of physiology and biochemistry of the exercise at the Physical Education School—UFPel. There was a maximum interval of 10 days between each visit. The participants were instructed to have a light meal two hours before each test and to avoid vigorous physical activity in the last 24 hours.

On the first visit, an incremental maximal cycle ergometer (Ergo-Fit 1200^®^) test was performed, following a modified Balke protocol [6]. Prior to the test, wearing only shorts and t-shirts, participants’ weight and height were measured using an electronic scale Soehnle Professional 7755^®^ (100 g precision) and a wall mounted stadiometer Stardard Sanny^®^ (1 mm precision), respectively. Among males, warm up consisted of pedalling at 100 watts for three minutes, followed by an increase to 150 watts, which was subsequently increased by 25 watts every minute. Among females, there was a warming up session during three minutes at 50 watts on the cycle ergometer, followed by an increase to 100 watts, which was subsequently increased in by 15 watts every minute. For both sexes, participants were instructed during the test to remain at the minimum frequency of 60 rotations per minute (rpm). The oxygen uptake was measured by a mixing-box-type portable gas analyser (VO2000, MedGraphics; Ann Arbor, USA) [7], previously calibrated according to manufacturer’s specifications. For every three breaths, one measure was performed, and the data were analysed using the BREEZE Software. Heart rate was assessed using a Polar V800^®^ monitor. Participants aged 45 years or older have their maximal heart rate (HR_max_) defined according to the following equation: HR_max_ = 208 –(0.7* Age) [8]. The tests were terminated by voluntary exhaustion or if participants reached their HR_max_.

In the second visit, participants were submitted to nine free-living activities (Table 1), based on a previous accelerometer calibration study [9]. The last and most intense activity was only performed by those who were willing. All activities lasted five minutes, except for the first one, which was based on 10 minutes supine. Among the first eight activities, there was a resting period of two minutes between each activity, and before the last activity there was a five-minute resting period due to an increase in the activity intensities. During all activities, the oxygen uptake was measured using the same procedures applied in the first visit.

**Table 1:** Description of the nine structured activities.

Order	Activity (minutes)	Description
1^st^	Lying down (10’)	Lying in supine position awake, with arms on the side, avoiding bodily movement.
2^nd^	Sitting (5’)	Sitting on a chair, using the computer.
3^rd^	Standing (5’)	Standing on the floor, using mobile phone.
4^th^	Circuit (5’)	Sitting, putting on shoes, standing, moving eight things on a desk, writing a message on a mobile phone, and sitting down again. Repeat.
5^th^	Slow walking, 3 km · h^-1^ (5’)	Walking on a treadmill.
6^th^	Brisk walking, 6 km · h^-1^ (5’)	Walking on a treadmill.
7^th^	Step (5’)	At the beginning of minute two and four, walking up a step (20 cm high) 15 times. The rest of the time, walking on the treadmill at 6 km.h^-1^.
8^th^	Running, 8 km · h^-1^ (5’)	Running on a treadmill.
9^th^	Intermittent running, 10 km · h^-1^ and 12 km · h^-1^ (5’)	Running at 10 km · h^-1^ for 60 seconds, alternating with running at 12 km · h^-1^ for 30 seconds on a treadmill.

### Analyses

Data reduction was performed to evaluate the period in which participants were in steady state in each activity. In the first activity, only the period between minutes 7 and 9.5 was evaluated and for the other activities the period between minutes 2.5 and 4.5 was assessed. After data reduction, an average of the oxygen uptake (ml·kg^-1^·min^-1^) of each activity was calculated and later converted to METs (1 MET = 3.5 ml·kg^-1^·min^-1^) [10]. The METs values were analysed as a continuous variable.

Criterion measure for physical activity intensities was classified according to current recommendations for exercise prescriptions by the *American College of Sports Medicine* [4]: percentage of maximal oxygen uptake (light, <46%; moderate, 46–63%; vigorous, ≥64%). These categories were dichotomized as (a) light *vs*. moderate to vigorous and (b) vigorous *vs*. lower than vigorous.

ROC curve analysis was performed to generate physical activity intensity thresholds in METs, according to the higher sensitivity (correctly identifying activities above the thresholds), specificity (correctly identifying activities below the thresholds) and area under the ROC curve (AUC). Similar analytical procedures were used elsewhere [11]. Additional analyses were carried out stratifying the sample by gender, age (20 to 39; and 40 to 60), body mass index (BMI) (normal: <25.0 kg/m^2^; and overweight/obese: ≥25.0 kg/m^2^) [12] and physical fitness, classified according to sex and age and categorized as low physical fitness (very bad, bad, below average and average) and high physical fitness (above average, good and excellent) [13]. The sample-size (using α = 0.05 and power = 80%) was sufficient to detect differences of 10 percentage points among AUC values. Comparisons between sensitivity, specificity and AUC from different thresholds were performed based on the range of interval values and overlapping 95% confidence intervals (CI) [14, 15]. Data analysis was carried out in *Stata12*.*1*.

## Results

A total of 103 participants attended the two visits. Among men, the average age was 36.1 (SD ± 11.1) years (two thirds of the participants were younger than 40 years old), 46% were classified with overweight and 26% presented above average physical fitness. Among women, most of the sample was younger than 40 years old (69.4%), classified as normal BMI (61.2%) and bad physical fitness (26.5%) (Table 2).

**Table 2:** Sample description according to demographic, nutritional and physiological variables.

Variables	Male		Female	
	N	%	N	%
**Age (years)**				
20–29	16	29.6	20	40.8
30–39	20	37.0	14	28.6
40–49	8	14.9	9	18.4
50–60	10	18.5	6	12.2
**BMI (kg/m**^**2**^**)**				
Normal (<25)	22	40.7	30	61.2
Overweight (25–29.9)	25	46.3	14	28.6
Obesity (≥30)	7	13.0	5	10.2
**Physical fitness**				
Very bad	0	0.0	6	12.2
Bad	1	1.9	13	26.5
Below average	10	18.5	12	24.5
Average	9	16.7	7	14.3
Above average	14	25.9	6	12.2
Good	8	14.8	4	8.2
Excellent	12	22.2	1	2.1
**Total**	**54**	**52.4**	**49**	**47.6**

The limits of physical fitness categories are expressed as maximal oxygen uptake (ml·kg^-1^·min^-1^) [13].

Mean values and standard deviation of the oxygen uptake for each activity are presented in Table 3. The mean oxygen uptake during the rest period (lying in supine position) was equal between men and women (1.0 (± 0.2) MET). Regarding the other activities, the means of oxygen uptake were also similar between men and women, except for brisk walking–women: 5.8 (± 1.1) METs; men: 5.0 (± 0.7) METs.

**Table 3:** Mean and standard deviation (SD) of oxygen uptake (ml·kg^-1^·min^-1^) and METs for each activity, stratified by sex.

Activities	Overall		Male		Female	
	VO_2_	METs	VO_2_	METs	VO_2_	METs
	Mean (±SD)	Mean (±SD)	Mean (±SD)	Mean (±SD)	Mean (±SD)	Mean (±SD)
1—Lying down	3.5 (±0.7)	1.0 (±0.2)	3.4 (±0.7)	1.0 (±0.2)	3.5 (±0.7)	1.0 (±0.2)
2—Sitting	4.5 (±0.9)	1.3 (±0.3)	4.4 (±0.9)	1.3 (±0.3)	4.6 (±0.9)	1.3 (±0.3)
3—Standing	4.3 (±1.0)	1.2 (±0.3)	4.4 (±1.1)	1.2 (±0.3)	4.2 (±0.9)	1.2 (±0.2)
3—Circuit	7.0 (±1.5)	2.0 (±0.4)	6.8 (±1.6)	1.9 (±0.5)	7.2 (±1.4)	2.1 (±0.4)
5—Slow walking, 3 km · h^-1^	10.6 (±2.0)	3.0 (±0.6)	10.1 (±1.8)	2.9 (±0.5)	11.2 (±2.2)	3.2 (±0.6)
6—Brisk walking, 6 km · h^-1^	19.0 (±3.5)	5.4 (±1.0)	17.7 (±2.4)	5.0 (±0.7)	20.4 (±3.9)	5.8 (±1.1)
7—*Step*	19.1 (±2.9)	5.5 (±0.8)	18.3 (±2.7)	5.2 (±0.8)	20.1 (±2.8)	5.7 (±0.8)
8—Running 8 km · h^-1^ (n = 101)	28.7 (±3.7)	8.2 (±1.1)	28.3 (±3.5)	8.1 (±1.0)	29.2 (±4.0)	8.3 (±1.1)
9—Intermittent running, 10 km · h^-1^ and 12 km · h^-1^ (n = 72)	36.5 (±5.0)	10.4 (±1.4)	36.4 (±5.1)	10.4 (±1.4)	36.9 (±4.9)	10.5 (±1.4)

n–Number of participants who performed the activities. Activities without this information were practiced by all participants.

The intensity thresholds identified in this study, based on the highest value sum in the sensitivity and specificity, were 4.9 METs for moderate and 6.8 METs for vigorous physical activity. Comparing these thresholds to those recommended by the current guidelines (≥3.0 METs for moderate and ≥6.0 METs for vigorous physical activity), we observed similar AUC. However, there were important differences in terms of sensitivity and specificity. The moderate threshold identified in the analytical sample was 1.9 METs higher compared to the recommended one, also presenting higher specificity (91.5; 95%CI: 88.9–93.6, compared to 78.8; 95%CI: 75.3–82.0, respectively). Regarding vigorous intensity thresholds, the estimate based on the analytical sample was 0.8 MET higher than the recommended value, presenting higher specificity as well (96.0; 95%CI: 94.3–97.3, compared to 92.1; 95%CI: 89.9–94.0) (Table 4).

**Table 4:** Physical activity intensity thresholds according to current physical activity guidelines and from this study (based on overall sample and stratified by sex, age, BMI and physical fitness variables).

Intensity thresholds		Sensitivity (%) (95%CI)	Specificity (%) (95%CI)	AUC (95%CI)
**Current guidelines**				
Moderate	3.0	100 (98.8–100)	78.8 (75.3–82.0)	0.89 (0.88–0.91)
Vigorous	6.0	83.1 (76.9–88.1)	92.1 (89.9–94.0)	0.88 (0.85–0.91)
**Overall sample**				
Moderate	4.9	92.4 (88.8–95.1)	91.5 (88.9–93.6)	0.92 (0.90–0.94)
Vigorous	6.8	77.2 (70.6–83.0)	96.0 (94.3–97.3)	0.87 (0.84–0.90)
**Sex**				
Male				
Moderate	5.6	85.8 (78.7–91.2)	96.8 (94.4–98.4)	0.91 (0.88–0.94)
Vigorous	7.2	98.7 (92.9–100)	93.8 (91.0–96.0)	0.96 (0.97–0.98)
Female				
Moderate	3.8	96.4 (92.4–98.7)	97.5 (94.6–99.1)	0.97 (0.95–0.99)
Vigorous	5.5	84.1 (76.0–90.3)	90.2 (86.2–93.3)	0.87 (0.83–0.91)
**Age**				
20–39 years				
Moderate	4.9	92.9 (88.5–96.0)	91.9 (88.8–94.4)	0.92 (0.90–0.95)
Vigorous	6.6	84.3 (76.7–90.1)	95.6 (93.4–97.3)	0.90 (0.87–0.93)
40–60 years				
Moderate	4.6	94.6 (87.8–98.2)	90.0 (84.8–93.9)	0.92 (0.89–0.95)
Vigorous	5.6	80.6 (68.6–89.6)	92.7 (88.5–95.8)	0.87 (0.81–0.92)
**BMI**				
Normal				
Moderate	5.0	93.0 (87.9–96.5)	91.1 (87.2–94.1)	0.92 (0.90–0.95)
Vigorous	7.0	80.8 (71.7–88.0)	96.3 (93.7–98.0)	0.89 (0.85–0.93)
Overweight/obesity				
Moderate	5.0	86.9 (80.3–91.9)	94.2 (90.9–96.6)	0.91 (0.88–0.94)
Vigorous	6.4	74.4 (64.2–83.1)	96.0 (93.4–97.8)	0.85 (0.81–0.90)
**Physical fitness**				
Low				
Moderate	4.0	96.3 (92.6–98.5)	94.0 (90.7–96.4)	0.95 (0.93–0.97)
Vigorous	6.4	70.4 (61.6–78.2)	99.2 (97.6–99.8)	0.84 (0.80–0.88)
High				
Moderate	6.2	92.9 (86.4–96.9)	99.0 (97.0–99.8)	0.96 (0.93–0.98)
Vigorous	8.2	92.2 (82.7–97.4)	95.8 (93.1–97.7)	0.94 (0.91–0.98)

Relative intensity thresholds based on percentage of maximal oxygen uptake (light, <46%; moderate, 46–63.9%; vigorous, ≥64%) were used as criterion measure.

Stratified intensity thresholds were also estimated and are presented in Table 4. All estimates for moderate intensity were higher than 3.0 METs. Among men, moderate physical activity threshold was 5.6 METs, while among women this threshold was 3.8 METs. Moderate thresholds of 4.0 and 6.2 METs were found when comparing participants with low and high physical fitness respectively. There were no or small differences in the moderate thresholds comparing BMI and age groups. Assessing vigorous physical activity intensity thresholds, two subgroups presented lower values compared to the recommended threshold (5.5 METs for women and 5.6 METs for participants between 40 and 60 years old). The higher threshold identified for vigorous physical activity was among participants with high physical fitness (8.2 METs). For all stratified analyses, AUC presented relatively high values, which was lower among participants with low physical fitness (AUC = 0.84; 95%CI: 0.80–0.88).

In Table 5, the overall thresholds identified in the analytical sample (4.9 METs for moderate and 6.8 METs for vigorous physical activity) were applied to each subgroup previously evaluated and, thereafter, sensitivity, specificity and AUC were calculated. Among men and participants with high physical fitness, despite not showing difference in terms of AUC, specificity from the specific moderate thresholds (96.8; 95%CI: 94.4–98.4 and 99.1; 95%CI: 97.0–99.8, respectively–Table 4) were higher compared to those based on overall estimates (86.4; 95%CI: 82.3–89.8 and 85.3; 95%CI: 80.7–89.2, respectively–Table 5). However, it was not identified for all other evaluated subgroups in terms of moderate thresholds. Regarding vigorous physical activity thresholds, differences were found only among women. The vigorous threshold, based on the overall sample, presented higher specificity (99.0; 95%CI: 97.1–99.8 –Table 5) than its specific threshold (90.2; 95%CI: 86.2–93.3 –Table 4). Moderate and vigorous intensity thresholds from the overall sample showed high AUC values when applied to specific groups, where the lowest values were identified among women and participants from 40 to 60 years old (0.81; 95%CI: 0.76–0.85 and 0.81; 95%CI: 0.75–0.87, respectively).

**Table 5:** Sensitivity, specificity and AUC from overall thresholds (4.9 METs for moderate and 6.8 METs for vigorous) applied to specific groups.

Sample groups	Intensity thresholds	Sensitivity (%) (95%CI)	Specificity (%) (95%CI)	AUC (95%CI)
**Male**				
	Moderate	94.0 (88.6–97.4)	86.4 (82.3–89.8)	0.90 (0.88–0.93)
	Vigorous	100 (95.3–100)	93.8 (91.0–96.0)	0.97 (0.96–0.98)
**Female**				
	Moderate	91.1 (85.8–94.9)	98.7 (96.4–99.7)	0.95 (0.93–0.97)
	Vigorous	61.9 (52.3–70.9)	99.0 (97.1–99.8)	0.81 (0.76–0.85)
**20–39 years**				
	Moderate	92.9 (88.5–96.0)	91.9 (88.8–94.4)	0.92 (0.90–0.95)
	Vigorous	82.7 (75.0–88.8)	96.0 (93.9–97.6)	0.89 (0.86–0.93)
**40–60 years**				
	Moderate	91.3 (83.6–96.2)	90.5 (85.4–94.3)	0.91 (0.87–0.95)
	Vigorous	66.1 (53.0–77.7)	95.9 (92.4–98.1)	0.81 (0.75–0.87)
**Normal BMI**				
	Moderate	95.6 (91.1–98.2)	89.7 (85.6–92.9)	0.93 (0.90–0.95)
	Vigorous	80.8 (71.7–88.0)	96.0 (93.4–97.8)	0.88 (0.84–0.92)
**Overweight/obesity BMI**				
	Moderate	89.0 (82.7–93.6)	93.2 (89.7–95.8)	0.91 (0.88–0.94)
	Vigorous	73.3 (63.0–82.1)	96.0 (93.4–97.8)	0.85 (0.80–0.89)
**Low physical fitness**				
	Moderate	88.0 (82.5–92.2)	97.3 (94.8–98.8)	0.93 (0.90–0.95)
	Vigorous	67.2 (58.2–75.3)	99.7 (98.5–100)	0.84 (0.79–0.88)
**High physical fitness**				
	Moderate	100 (96.8–100)	85.3 (80.7–89.2)	0.93 (0.91–0.95)
	Vigorous	96.9 (89.2–99.6)	91.9 (88.5–94.6)	0.94 (0.92–0.97)

## Discussion

The present study evaluated validity parameters of thresholds based on absolute physical activity intensities (expressed in METs) according to the current guidelines [3], and original thresholds using relative intensities as criterion measure. Our results indicated higher thresholds for moderate (4.9 METs) and for vigorous activity (6.8 METs) than current recommended thresholds found in the literature.

A necessary discussion to interpret our results is regarding the most adequate criterion measure to define light, moderate and vigorous physical activity. It is important to highlight the absence of a gold standard to classify physical activity intensities and, therefore, absolute or relative intensities were applied. These two methods are highly correlated to define time spent in different physical activity intensities and might be similar across laboratorial studies based on a homogeneous sample in terms of sex, age and physical fitness. Nevertheless, considering population-based samples (higher heterogeneity), absolute intensities might result in misclassification and wider differences between absolute and relative thresholds [16].

Thereafter, absolute thresholds were presented according to an adequate analytical process, in which the criterion measure consisted in categories of relative intensity. The thresholds were identified according to the greatest sum between sensitivity and specificity and, consequently, with the highest accuracy. Although no difference in terms of accuracy was identified comparing our overall thresholds to the recommended one, there were differences in the sensitivity and specificity parameters.

Absolute intensity thresholds have been widely applied in epidemiology association-based studies and also used as criterion measure in calibration studies of questionnaires and accelerometers. However, it might not be the most adequate procedure. Esliger *et al* (2011) [11], discussed that absolute thresholds currently recommended could be lower the correct intensity classification. In their study, calibration analyses used 4.0 METs and 7.0 METs to classify the criterion measure as moderate and vigorous physical activity, respectively.

Using lower intensity thresholds, which usually present higher sensitivity, but lower specificity and accuracy, misclassification in terms of physical activity will be likely higher. For example, applying the widely recommended thresholds proposed in 1995 [17], an activity such as walking slowly (≤2.0 mph or ≤3.2 km · h^-1^) will be considered as a light physical activity, presenting an oxygen uptake lower than 3.0 METs. However, Esliger *et al* (2011) [11], identified an average oxygen uptake of 3.9 (± 0.7) METs for a slightly faster walking (4.0 km · h^-1^), which exceeded almost 1.0 MET the recommended threshold. In the present analyses, the average oxygen uptake for a 3.0 km · h^-1^ walking was 3.0 (± 0.6) METs, similar to Esliger *et al* (2011) [11], which would be considered as a moderate physical activity according to the current guidelines [17, 3].

Considering the direct relationship between benefits and intensities of physical activities [3], lower intensity thresholds with lower specificity, such as the recommended ones, might overestimate moderate physical activity practice, by including light physical activities in this category. This misclassification may attenuate physical activity effects on health outcomes, such as mental health and hypertension, which are associated mostly to moderate physical activity [16]. Furthermore, overestimation of vigorous physical activity might bias the effect of physical activity on cardiovascular diseases and osteoporosis, for example, which is mostly influenced by this physical activity intensity [16].

Group-specific thresholds were also presented in this study due to the possible influence of sex, age, nutritional status and physical fitness on physical activity intensity thresholds. Our hypothesis was that group-specific thresholds would present higher accuracy. However, most group-specific analyses refuted such hypothesis (Tables 4 and 5). Differences in sensitivity and specificity parameters were identified only among men and women, and among participants with high physical fitness. In these groups, the use of specific thresholds could be considered a useful alternative in comparison to the application of overall thresholds (based on the complete analytical sample).

Some limitations must be considered to interpret the present results. The sample was selected by convenience and included only healthy participants. Although the sample was composed of participants with different characteristics that could influence physical activity intensities (sex, age, nutritional status and physical fitness), our sample should not be considered representative of a general adult population.

Furthermore, the applied protocol was restricted to nine activities, which represent some, but not all free-living activities. On the other hand, the activities chosen might be considered representative of most adult activities during the awake period.

The physical fitness measure analysed was the peak oxygen uptake, however, these measures were grouped using classifications related to percentage of maximal oxygen uptake instead of percentage of peak oxygen uptake. This analysis criterion was adopted due to the fact that oxygen uptake classifications were based on maximal oxygen uptake [4]. Considering that peak oxygen uptake is a valid predictor of maximal oxygen uptake [18, 19, 20], we believe that this methodological procedure was adequate, without compromising the validity of the obtained results.

Oxygen uptake measurements were obtained using a cycle ergometer instead of a treadmill exercise protocol. We are aware that oxygen uptake values obtained in cycle ergometer and treadmill may be different as cycling is not a regular exercise for most individuals and fewer muscles are used during this exercise [21]. Nevertheless, in the general population, as it is the case of our sample, the difference between oxygen uptake values from treadmill and cycle ergometer tests is lower than 10% [22]. Thus, we strongly believe that our results would not be different if another ergometer was used. Another important issue to be highlighted is that none of the participants were regular cyclists but all were familiarized with riding a cycle ergometer, decreasing the chance of differential errors among the estimates. Furthermore, the participants sampled were not familiar with walking/running in the treadmill and thus, some familiarization sessions would be required prior data collection, increasing participant’s burden and risking drop out of the study. In this context, cycling on an ergometer was easier and safer when conducting tests to exhaustion.

The oxygen uptake reserve was not considered for the analyses. However, it would not imply relevant differences from our results, since the resting oxygen uptake values were very similar among the individuals. This approach is in accordance with other studies [12, 23, 24].

Considering as strength of the present study, the oxygen uptake measurement was performed using indirect calorimetry, which is considered a gold standard to evaluate oxygen uptake in laboratorial settings [25, 26]. Finally, another strength is the relatively large sample size analysed for a calibration study with complex physical activity protocol. More than a hundred participants had their peak oxygen uptake evaluated and completed the research protocol, allowing the use of relative physical activity intensities as a criterion measure for the identification of absolute intensity thresholds in METs.

## Conclusions

The physical activity thresholds generated for the entire sample (moderate: 4.9 METs; vigorous: 6.8 METs) were not chosen arbitrarily, but were created following methodological criteria appropriate to this objective and using categories of relative intensity for each participant as a criterion measure. As a result, the identified intensity thresholds were higher and presented higher specificity when compared to thresholds currently recommended. The use of the proposed thresholds in this study aims to improve the quality of physical activity measure, minimizing errors in the evaluation of physical activity intensities. Moreover, these parameters presented relatively high accuracy, including when specifically applied to groups of sex, age, nutritional status and physical fitness. Therefore, the overall thresholds, as well as those related specifically to men and women, might be an important alternative to minimize physical activity intensity misclassification. The results presented in this study contribute towards more accurate physical activity measure and highlight the relevance of a better understanding regarding the impact of physical activity intensity thresholds in health outcomes.
